# ICIs治疗晚期非小细胞肺癌免疫相关不良事件的系统综述及*Meta*分析

**DOI:** 10.3779/j.issn.1009-3419.2020.104.07

**Published:** 2020-09-20

**Authors:** 侨西 秦, 稼劲 王, 红 王

**Affiliations:** 1 100071 北京，解放军总医院第五医学中心肺部肿瘤科 Deparment of Lung Cancer, the Fifth Medical Center, General Hospital of People's Liberation Army, Beijing 100071, China; 2 100036 北京，解放军总医院第六医学中心消化内科 Department of Gastroenterology, the Sixth Medical Center, General Hospital of People's Liberation Army, Beijing 100036, China

**Keywords:** 免疫检查点抑制剂, 肺肿瘤, 免疫相关不良反应, *Meta*分析, Immune Checkpoint Inhibitors, Lung neoplasms, Immune-related adverse events, *Meta*-analysis

## Abstract

**背景与目的:**

近年来，免疫检查点抑制剂(immune checkpoint inhibitors, ICIs)因其对非小细胞肺癌(non-small cell lung cancer, NSCLC)患者在延长生存方面显示出了显著的疗效而成为肿瘤领域的热点。但其介导的免疫相关不良事件(immune-related adverse events, ir-AEs)由于其特异性、严重性亦频发报道。本研究拟系统评价ICIs治疗晚期NSCLC患者所致ir-AEs，旨在为晚期NSCLC患者的治疗选择及ir-AEs的早期诊断和治疗提供参考。

**方法:**

检索The Cochrane Library、PubMed、EMBASE等数据库关于ICIs治疗晚期NSCLC的随机对照试验(randomized controlled trial, RCT)。主要结局指标包括ir-AEs发病数量、级别。采用相对危险度(relative risk, RR)为效应量，各效应量以95%置信区间(confidence interval, CI)表示; 应用Stata 15.0/Revman 5.3软件进行*meta*分析。

**结果:**

本文共纳入17个RCTs。ICIs所致ir-AEs一般多于传统化疗组。在ICIs单药治疗组中，抗细胞毒性T淋巴细胞相关抗原4(cytotoxic T-lymphocyte-asscociated antigen 4, CTLA-4)组所致ir-AEs的总体发病率最高; ICIs联合治疗所致各级别ir-AEs的发病率高于单药治疗组，但其所致严重ir-AEs的发病率与抗CTLA-4组相似。

**结论:**

ICIs与传统化疗毒性谱不同，其免疫相关毒性较传统化疗更强。ICIs所致ir-AEs具有器官特异性，不同类型的ICIs具有独特的免疫相关毒性谱。随着ICIs逐渐成为治疗晚期NSCLC的一种新型、有效的治疗方案，本文通过系统阐述ICIs治疗晚期NSCLC所致ir-AEs，为临床医生和患者对ICIs进一步的认识提供了帮助，并对ir-AEs的早发现、早诊断、早治疗提供数据参考及管理建议，使晚期NSCLC患者进一步从免疫疗法中获益。

## Abstract (en)

**Background and objective:**

In recent years, immune checkpoint inhibitors (ICIs) have become a hot spot in cancer because of their remarkable survival benefits on non-small cell lung cancer (NSCLC) patients. However, the immune-related adverse events (ir-AEs) induced by ICIs have been frequently reported due to its specificity and severity. This article is to summarize and evaluate ir-AEs induced by ICIs. Hopefully it can provide guidance for advanced NSCLC patients treatment options, early recognition and management of ir-AEs.

**Methods:**

Randomized controlled trials (RCT) which involved ICIs in the treatment of advanced NSCLC were retrieved in the Cochrane Libraby, PubMed, EMBASE and other databases. The primary outcome includes the incidence, grade and organ specificity of ir-AEs. Relative risk (RR) was used as the effect size, which was expressed as 95% confidence interval (CI). The Stata 15.0 and RevMan 5.3 software are used to conduct the meta analysis.

**Results:**

A total of 17 RCTs were included. The ir-AEs were generally more than those in the traditional chemotherapy group. The risk and severity of ir-AEs induced by ICIs combined group were generally higher than that of ICI monotherapy, while the incidence of severe ir-AEs induced by ICIs combination therapy was similar to that of anti-cytotoxic T-lymphocyte-asscociated antigen 4 (CTLA-4) group.

**Conclusion:**

ICIs have different toxicity profile compared with chemotherapy, and their immune-related toxicity is stronger than that of traditional chemotherapy. ICIs induced ir-AEs is organ-specific, and different ICI has specific immune-related toxicity profiles. As ICIs represent a new and distinct class of treatment for NSCLC, this article has systematically illustrated the efficacy and ir-AEs induced by ICIs, hopefully it can be useful for clinicians and patients to get a further understanding of ICIs, and facilitate early prediction, comprehensive diagnosis, and prompt management of ir-AEs by providing status reference and management suggestions, so that ICI can bring more benefit for advanced NSCLC patients.

根据2019癌症数据报告，肺癌仍是目前人类致死率最高(男性24%，女性23%)的肿瘤^[1]^。非小细胞肺癌(non-small cell lung cancer, NSCLC)约占所有肺癌组织类型的80%-85%，其中腺癌(约占40%-50%)和鳞状细胞癌(约占20%-30%)是NSCLC的主要组织学亚型^[2]^。但临床上往往只有一小部分NSCLC患者能够在早期被诊断，超过60%的患者在发现时已出现局部或远隔器官的转移，因此难以行根治性手术治疗，常规化疗和放疗是过去几十年中肺癌治疗的主要手段。近年来，针对程序性死亡受体1(programmed death 1, PD-1)及其配体(programmed death ligand 1, PD-L1)和细胞毒性T淋巴细胞相关抗原4(cytotoxic T-lymphocyte-asscociated antigen 4, CTLA-4)的免疫检查点抑制剂(immune checkpoint inhibitors, ICIs)的研究因其对NSCLC、黑色素瘤、肾细胞癌等在延长患者生存方面显示出了显著的疗效而成为肿瘤领域的热点。

目前，美国食品药品管理局(Food and Drug Administration, FDA)批准的ICIs主要通过阻断PD-1/PD-L1、CTLA-4信号通路，解除肿瘤细胞的免疫耐受状态，从而产生抗肿瘤效应。但由于其非特异性的正向免疫调节作用，ICIs介导的免疫相关不良反应(immune-related adverse events, ir-AEs)如肺炎^[3]^、甲状腺功能减退^[4]^等，由于其特异性、严重性亦频发报道。这些不良反应涉及皮肤、消化系统、呼吸系统、泌尿系统、内分泌及代谢系统、骨骼及肌肉等人体的诸多方面。因ICIs的种类不同，其介导ir-AEs机制也有所不同，因此我们拟对PD-1/PD-L1、CTLA-4抗体治疗晚期NSCLC患者所致ir-AEs做一系统回顾及*meta*分析，旨在为ir-AEs的早期诊断和治疗提供参考。

## 1 资料与方法

### 1.1 检索策略

#### 1.1.1 数据库

两名研究者按照主题词与自由词相结合的检索方法分别检索PubMed、EMBASE、The Cochrane Library等数据库正文内容。①检索抗PD-1、PD-L1、CTLA-4对比传统化疗治疗晚期NSCLC的随机对照实验(randomized controlled trials, RCTs)，用于比较ICIs与传统化疗所致ir-AEs的发病率、级别、器官特异性。②检索不同种类ICIs联合及单药治疗晚期NSCLC的临床试验，用于比较ICIs联合用药与单药治疗以及不同种类ICIs所致ir-AEs的发病率、级别、器官特异性。

#### 1.1.2 检索式

正文内容检索式的制定根据PICOS原则，采用主题词与自由词相结合的方式，将同部分的检索词用“OR”连接，将不同部分的检索词用“AND”连接，同时扩大检索纳入文献的参考文献。各部分的检索对象如下：①研究对象(types of participants)：为晚期NSCLC; ②干预措施(types of interventions)：为PD-1/PD-L1、CTLA-4相关的免疫检查点抑制剂，包括“nivolumab”、“pembrolizumab”、“atezolizumab”、“durvalumab”、“avelumab”、“ipilimumab”、“tremelimumab”等; ③对照措施(types of comparisons)：为传统化疗; ④结局指标(types of outcomes)：包括ir-AEs的发病率、ir-AEs的级别; ⑤研究设计类型(types of studies)：为RCTs。仅以PubMed为例列举检索策略(图 1)。

**1:**
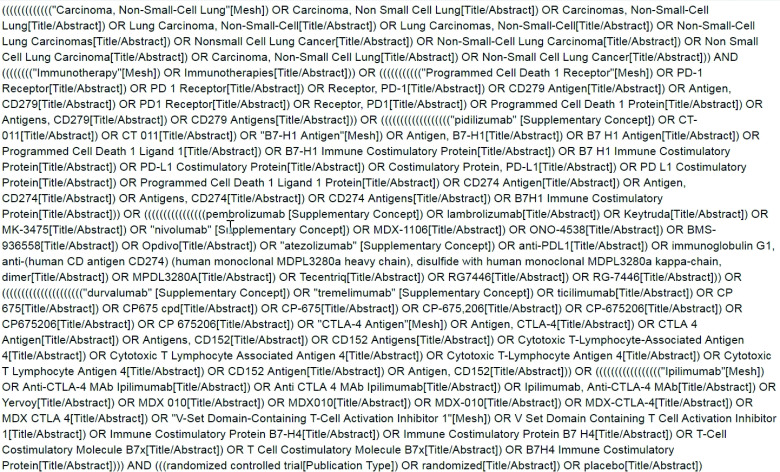
PubMed检索式 The PubMed search strategy

#### 1.1.3 检索时间及语种

检索时间截止到2019年12月。检索无语种限制。

### 1.2 纳入标准

#### 1.2.1 研究对象

年龄≥18岁，病理证实为晚期NSCLC患者，无绝对免疫治疗禁忌症。

#### 1.2.2 干预措施

实验组采用抗PD-1、抗PD-L1、抗CTLA-4免疫治疗，对照组采用化疗等标准治疗。

#### 1.2.3 研究类型

公开发布的前瞻性随机对照试验。

#### 1.2.4 结局指标

纳入文献需提供如下结局指标：全身及器官特异性各级别ir-AEs数量、全身及器官特异性3级及以上ir-AEs数量。

### 1.3 排除标准

综述、病例报道、会议摘要、非随机对照试验、合并其他恶性肿瘤以及数据无法进行分析的研究。

### 1.4 文献筛选和数据提取

两名研究者按照文献的纳入和排除标准，首先对问题和摘要进行初筛，其次通过阅读全文进行排除，意见不统一时通过讨论或第三方解决。提取信息主要包括：①一般信息：题目、第一作者、发表年份、试验设计类型、试验阶段、受试者人数。②研究对象的临床特征：性别、年龄、分期、病理类型、吸烟状况、PD-L1表达状况等。③干预措施：免疫治疗方案(ICIs类型、药品名、剂量，联合用药类型、药品名、剂量)、化疗方案(药品名、剂量)。如果该试验为多臂研究(如，Herbst R.S.2016试验组包含pembrolizumab 2 mg/kg组及10 mg/kg组)，我们则将各ICIs干预组分别与对照组相比较。④结局指标：全身及器官特异性各级别ir-AEs发病率、全身及器官特异性3级及以上ir-AEs发病率。

### 1.5 质量评价

依据Cochrane系统评价手册5.1.0质量评价标准从随机分配方法(random sequence generation)、分配隐藏(allocation concealment)、盲法(blinding of participants and personnel, blinding of outcome assessment)、不完整数据报告(incomplete outcome data)、选择性发表(selective reporting)及其他偏倚来源(other bias)等6个方面对纳入文献交叉进行质量评价。对拟纳入的每一项研究，分别按照上述6条标准，做出“低风险”(low risk)、“风险未知”(unclear)和“高风险”(high risk)的判断。评价结果含4条及以上低风险者，其发生各种偏倚的可能性最小，质量为A级; 含2条-3条低风险者，有发生相应偏倚的中度可能性，质量为B级; ≤上述1条低风险者有发生相应偏倚的高度可能性，质量为C级。

### 1.6 统计学方法

采用Stata 15.0/Revman 5.3软件进行。分别计算免疫治疗组与对照组ir-AEs发病率的相对危险度(relative risk, RR)及其95%置信区间(confidence interval, CI)。各组ir-AEs发病率的RR根据发生ir-AEs患者的绝对人数计算。ir-AEs的RR > 1表明试验组的免疫相关毒性更高。*P* < 0.05则差异有统计学意义。采用*Cochrane Q*检验进行纳入研究间的统计学异质性检测，同时采用*I*^2^作为统计量来描述研究间由于异质性而非偶然性而导致的总变异百分比从而评价异质性的大小(0%表示没有观察到异质性，而0%和100%之间的值表示异质性增加)。当*I*^2^ < 50%时，采用固定效应模型，反之则采用随机效应模型。如果异质性明显，我们通过不同类型的ICIs、研究对象PD-L1表达水平等方面进行亚组分析，从而探索异质性来源，必要时可采用敏感性分析以检验结果的稳定性。若异质性过大无法进行*meta*分析，则只做一般性描述。

## 2 结果

### 2.1 文献检索结果及质量评价

本研究共检索到1, 531篇相关文献。首先排除重复文献464篇，再经阅读标题和摘要排除综述、病例报道、*meta*分析、会议摘要、动物实验、非随机对照试验等共1, 030篇，最后经阅读全文从而排除数据不完整的RCTs，共17项研究最终纳入本*meta*分析，因检索到PD-L1联合CTLA-4治疗晚期NSCLC的RCT^[5]^在本文检索截止日期前尚未提供完整数据报告，故本*meta*分析对PD-L1联合CTLA-4治疗晚期NSCLC所致ir-AEs仅做一般性描述。文献的筛选流程如图 2所示。本文共纳入17项研究，其不良事件数据均在ClinicalTrials.gov可见。纳入文献的基本特征如表 1所示，其中3个为多臂研究(多臂研究的各ICIs干预组分别与对照组相比较)。ICIs治疗组共20个，其中抗PD-1组12个、抗PD-L1组4个、抗CTLA-4组3个、抗PD-1联合抗CTLA-4组1个; 传统化疗组17个。对ir-AEs的评估基于不良事件的通用术语标准(Common Terminology Criteria for Adverse Events) 3.0或4.0版本(皮疹的分级是两个版本之间的主要差异)。

**2:**
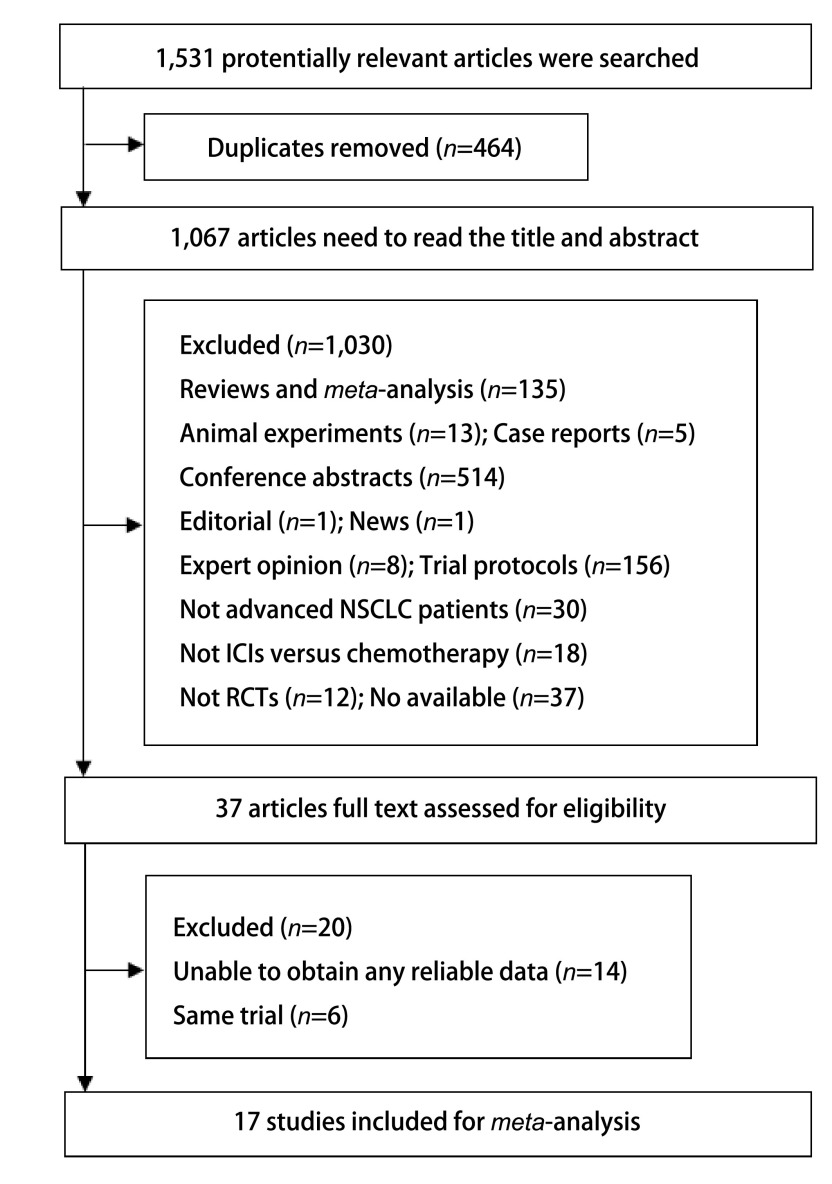
文献筛选流程图 Study identification and selection process

**1:** 纳入文献的基本特征 The characteristics of included studies

Study/Year	NCT no.	Design	Population	Size	Histology	PD-L1 cut-off	Target	ICIs	Control
Borghaei H. 2015^[6]^	NCT01673867	RCT/Phase 3	Stage IIIb/IV or recurrent non-squamous NSCLC	582	Non-squamous	1%, 5%, 10%	PD-1	Nivolumab	Docetaxel
Brahmer J. 015^[7]^	NCT02041533	RCT/Phase 3	Stage IIIb/IV squamous NSCLC	272	Squamous	1%, 5%, 10%	PD-1	Nivolumab	Docetaxel
Carbone D.P. 2017^[8]^	NCT02041533	RCT/Phase 3	Untreated stage IV or recurrent NSCLC, PD-L1≥1%	423	Any	1%, 5%	PD-1	Nivolumab	Chemotherapy
Wu Y.L. 2019^[9]^	NCT02613507	RCT/Phase 3	NSCLC that had progressed after platinum-based doublet chemotherapy	504	Any	1%	PD-1	Nivolumab	Docetaxel
Gandhi L. 2018^[10]^	NCT02578680	RCT/Phase 3	Metastatic non-squamous NSCLC without sensitizing EGFR or ALK mutations	616	Non-squamous	1%, 50%	PD-1	Pembrolizumab	Platinum-based chemotherapy
Herbst R.S. 2016^[11]^	NCT01905657	RCT/Phase 2/3	≥1% PD-L1 expression previously treated NSCLC	991	Any	1%, 50%	PD-1	Pembrolizumab	Docetaxel
Langer C. 2016^[12]^	NCT02039674	RCT/Phase 2	Untreated IIIb/IV non-squamous NSCLC without sensitizing EGFR or ALK mutations	123	Non-squamous	1%, 50%	PD-1	Pembrolizumab	Pemetrexed/ Carboplatin
Mok T.S.K. 2019^[13]^	NCT02220894	RCT/Phase 3	Previously treated locally advanced or metastatic NSCLC, PD-L1 TPS≥1%	1, 274	Any	1%, 20%, 50%	PD-1	Pembrolizumab	Platinum-based chemotherapy
Paz-Ares L. 2018^[14]^	NCT02775435	RCT/Phase 3	Untreated metastatic squamous NSCLC	559	Squamous	1%, 50%	PD-1	Pembrolizumab	Chemotherapy
Reck M. 2016^[15]^	NCT02142738	RCT/Phase 3	Untreated NSCLC, PD-L1≥50%, without sensitizing EGFR or ALK mutations	305	Any	50%	PD-1	Pembrolizumab	Platinum-based chemotherapy
Antonia S. 2017^[16]^	NCT02125461	RCT/Phase 3	Stage III NSCLC without disease progression after chemoradiotherapy	709	Any	-	PD-L1	Durvalumab	Chemotherapy
Fehrenbacher L. 2016^[17]^	NCT01903993	RCT/Phase 2	Progressed on post-platinum chemotherapy NSCLC	277	Any	1%, 5%, 50%	PD-L1	Atezolizumab	Docetaxel
Rittmeyer A. 2017^[18]^	NCT02008227	RCT/Phase 3	Previously treated IIIb/IV NSCLC	850	Any	1%, 5%, 50%	PD-L1	Atezolizumab	Docetaxel
Barlesi F. 2018^[19]^	NCT02395172	RCT/Phase 3	Stage IIIb or IV or recurrent NSCLC	792	Any	1%	PD-L1	Avelumab	Docetaxel
Lynch T. 2012^[20]^	Not Reported	RCT/Phase 2	chemotherapy-naïve IIIb/IV NSCLC	204	Any	-	CTLA-4	Ipilimumab	Chemotherapy
Govindan R. 2017^[21]^	NCT01285609	RCT/Phase 3	Stage IV or recurrent chemotherapy-naïve squamous NSCLC	749	Squamous	-	CTLA-4	Ipilimumab	Chemotherapy
Hellmann M. 2019^[22]^	NCT02477826	RCT/Phase 3	Stage IV or recurrent NSCLC previously untreated with chemotherapy	1739	Any	1%	PD-1/ CTLA-4	Nivolumab/ Ipilimumab	Chemotherapy
NSCLC: non-small cell lung cancer; EGFR: epidermal growth factor receptor; ALK: anaplastic lymphoma kinase; RCT: randomized controlled trial; PD-L1: programmed death ligand 1; PD-1: programmed death 1; CTLA-4: cytotoxic T-lymphocyte-asscociated antigen 4, CTLA-4.									

本文所纳入的17项研究均为随机对照实验，故所有或大部分纳入研究的测量偏倚、报告偏倚及其他偏倚较低; 而部分研究因缺乏部分失访、退出人群的部分结局指标故失访偏倚较高; 在选择偏倚上，由于部分研究的信息不足，无法评价其随机序列的产生和分配隐藏方法。根据Cochrane风险偏倚评估工具，利用Revman 5.3软件对所纳入的17项研究进行风险偏倚评价如图 3、图 4所示，本研究主要存在：①选择性偏倚：8项研究未描述随机序列产生的具体方法和分配隐藏方案的细节; ②失访偏倚：只有3项研究报道了失访和退出人群的次要结局指标。

**3:**
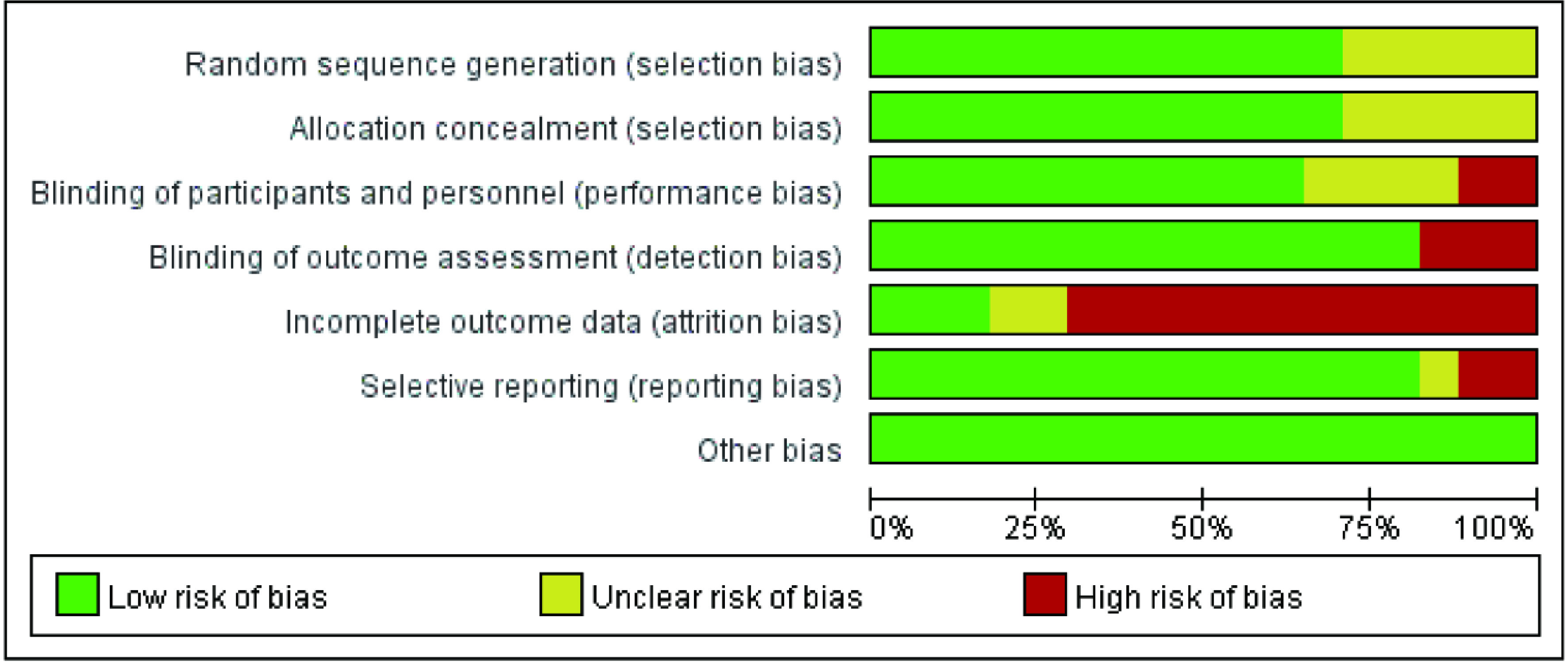
纳入研究的偏倚风险评价 Risk of bias assesment of included studies

**4:**
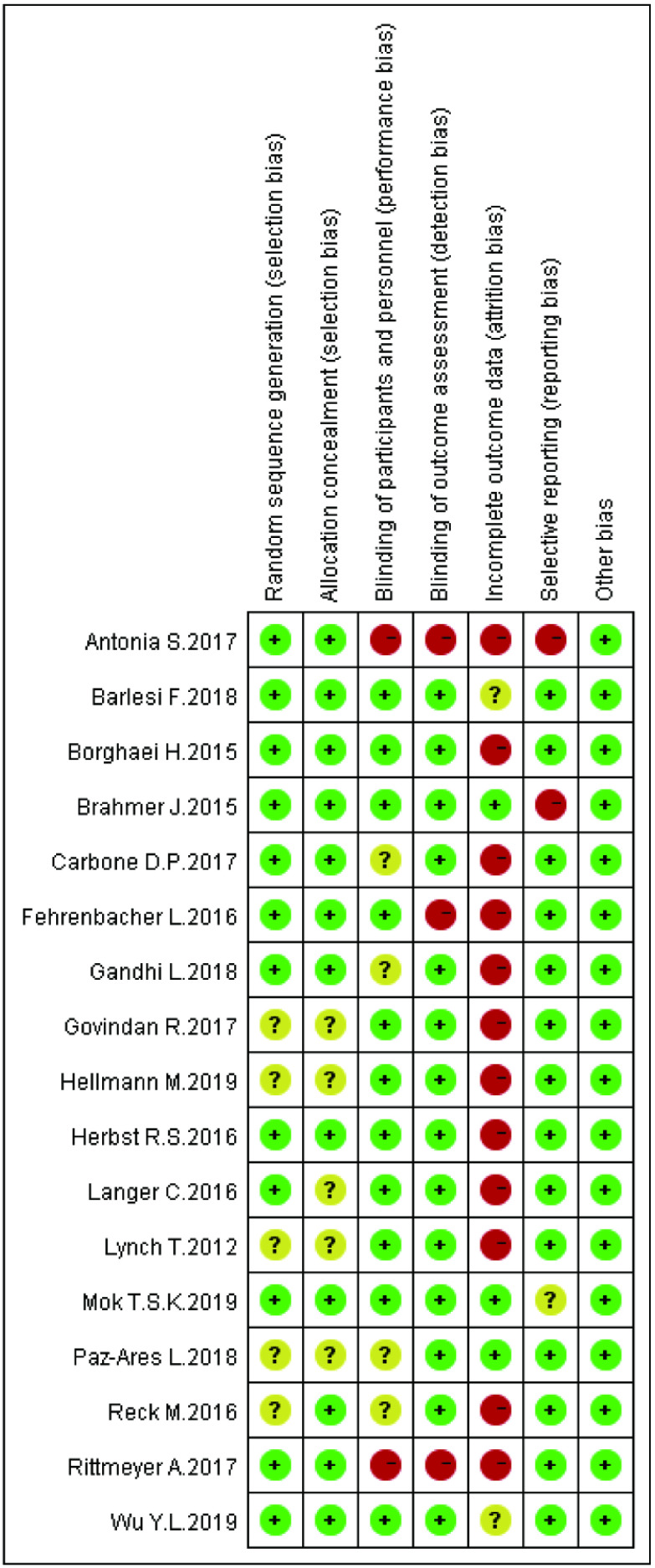
纳入研究的偏倚风险评价总结 The summary of risk of bias assesment of included studies

### 2.2 *Meta*分析结果

#### 2.2.1 ICIs对比传统化疗所致ir-AEs的发病风险

如图 5-图 11所示，本研究通过Revman 5.3软件计算了ICIs与传统化疗所致各器官ir-AEs的相对危险度(relative risk, RR)。我们观察到与传统化疗相比，ICIs组发生所有级别ir-AEs的风险总体较高，其中尤以发生内分泌ir-AEs的风险最高(RR 7.73, 95%CI: 4.89-12.20, *P* < 0.000, 01)，其次依次为肺脏(RR 4.46, 95%CI: 2.67-7.45, *P* < 0.000, 01)、皮肤(RR 2.41, 95%CI: 1.86-3.11, *P* < 0.000, 01)、肝脏(RR 2.33, 95%CI: 1.95-2.80，*P* < 0.000, 01)、肾脏(RR 1.59, 95%CI: 1.13-2.22, *P*=0.007)，而胃肠道ir-AEs及过敏反应的发生风险并无明显增加，RR分别为1.17(95%CI: 0.78-1.76, *P*=0.44)、0.78(95%CI: 0.60-1.01, *P*=0.06)。对于严重(≥3级)ir-AEs，ICIs组相较于传统化疗组发生风险最高的为内分泌(RR=8.84, 95%CI: 4.73-16.53, *P* < 0.000, 01)，其次为肝脏(RR=6.93, 95%CI: 4.25-11.29, *P* < 0.000, 01)、皮肤(RR =3.89, 95%CI: 2.52-6.01, *P* < 0.000, 01)、胃肠道(RR=3.87, 95%CI: 2.69-5.58, *P* < 0.000, 01)、肺脏(RR=3.39, 95%CI: 2.29-5.02, *P* < 0.000, 01)、肾脏(RR=3.39, 95%CI: 1.66-6.93, *P*=0.000, 8)，而严重级别过敏反应的发生风险仍无明显增加(RR=1.62, 95%CI: 0.97-2.69, *P*=0.07)。

**5:**
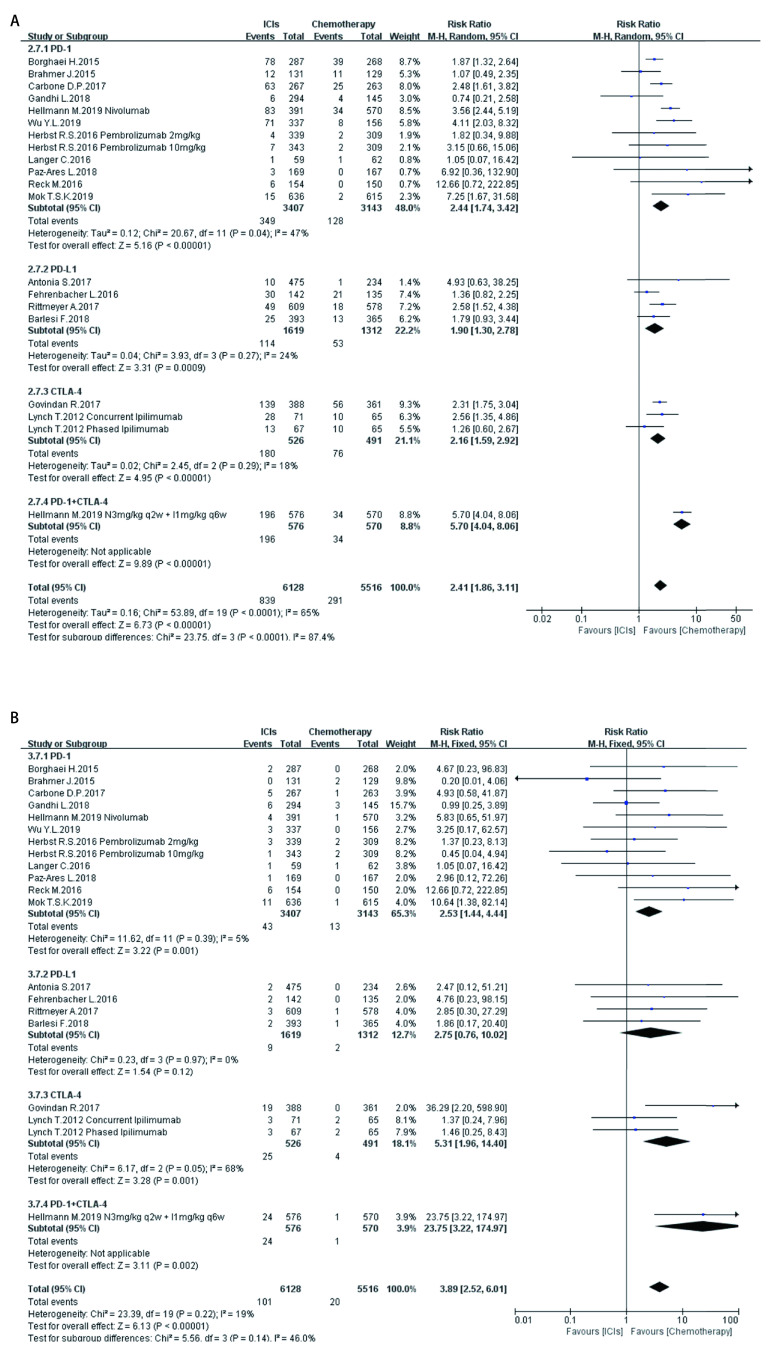
ICIs对比传统化疗所致皮肤ir-AEs。A：各级别皮肤ir-AEs; B：严重皮肤ir-AEs。 Skin ir-AEs induced by ICIs versus chemotherapy. A: Any grade skin ir-AEs; B: High grade skin ir-AEs

**6:**
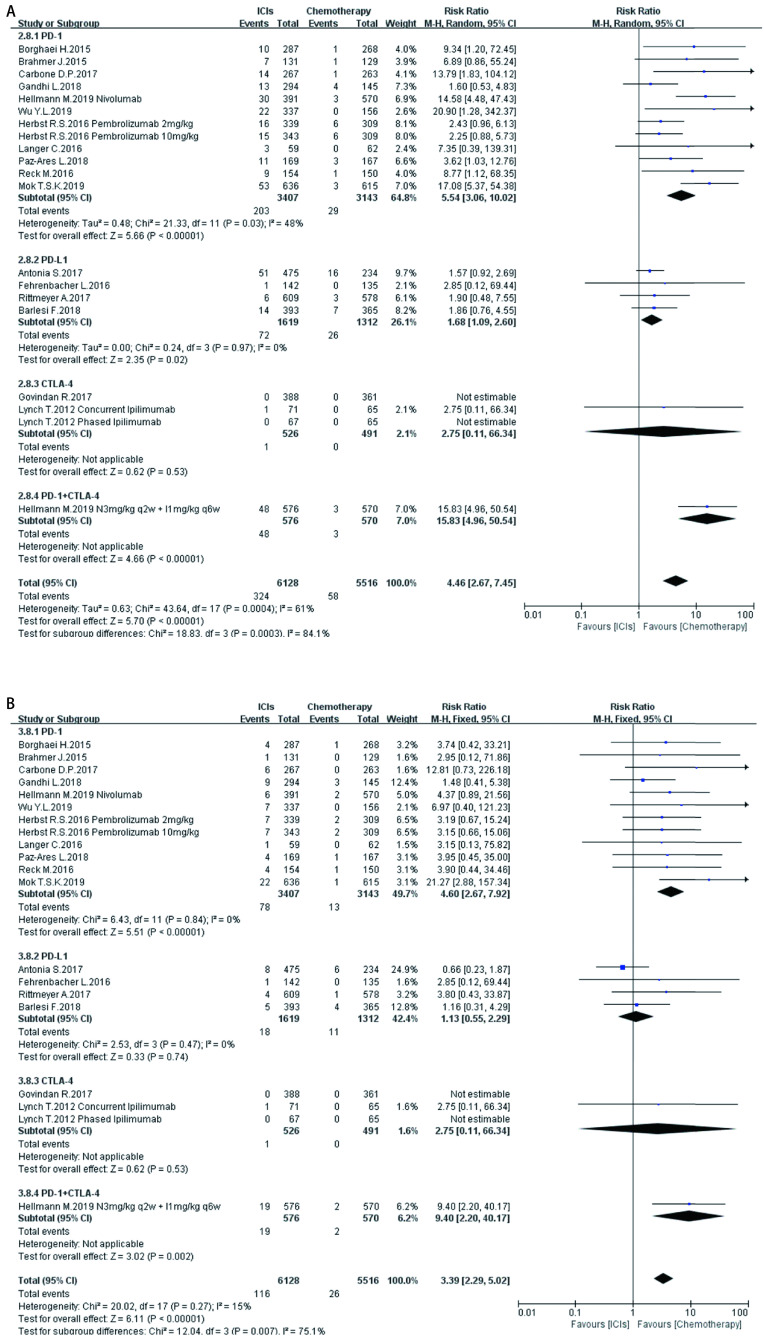
ICIs对比传统化疗所致肺脏ir-AEs。A：各级别肺脏ir-AEs; B：严重肺脏ir-AEs。 Pulmonary ir-AEs induced by ICIs versus chemotherapy. A: Any grade pulmonary ir-AEs; B: High grade pulmonary ir-AEs.

**7:**
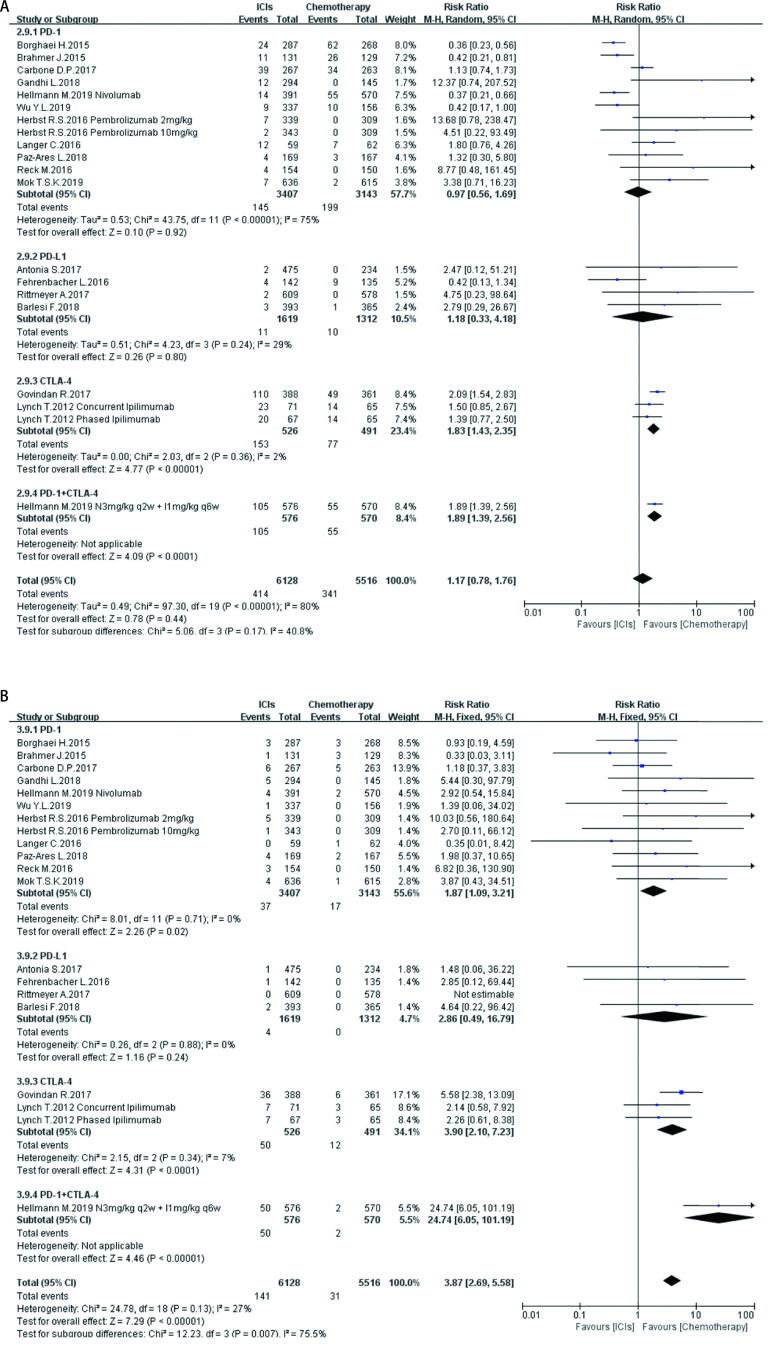
ICIs对比传统化疗所致胃肠道ir-AEs。A：各级别胃肠道ir-AEs; B：严重胃肠道ir-AEs。 Gastrointestinal ir-AEs induced by ICIs versus chemotherapy. A:Any grade gastrointestinal ir-AEs; B: High grade gastrointestinal ir-AEs.

**8:**
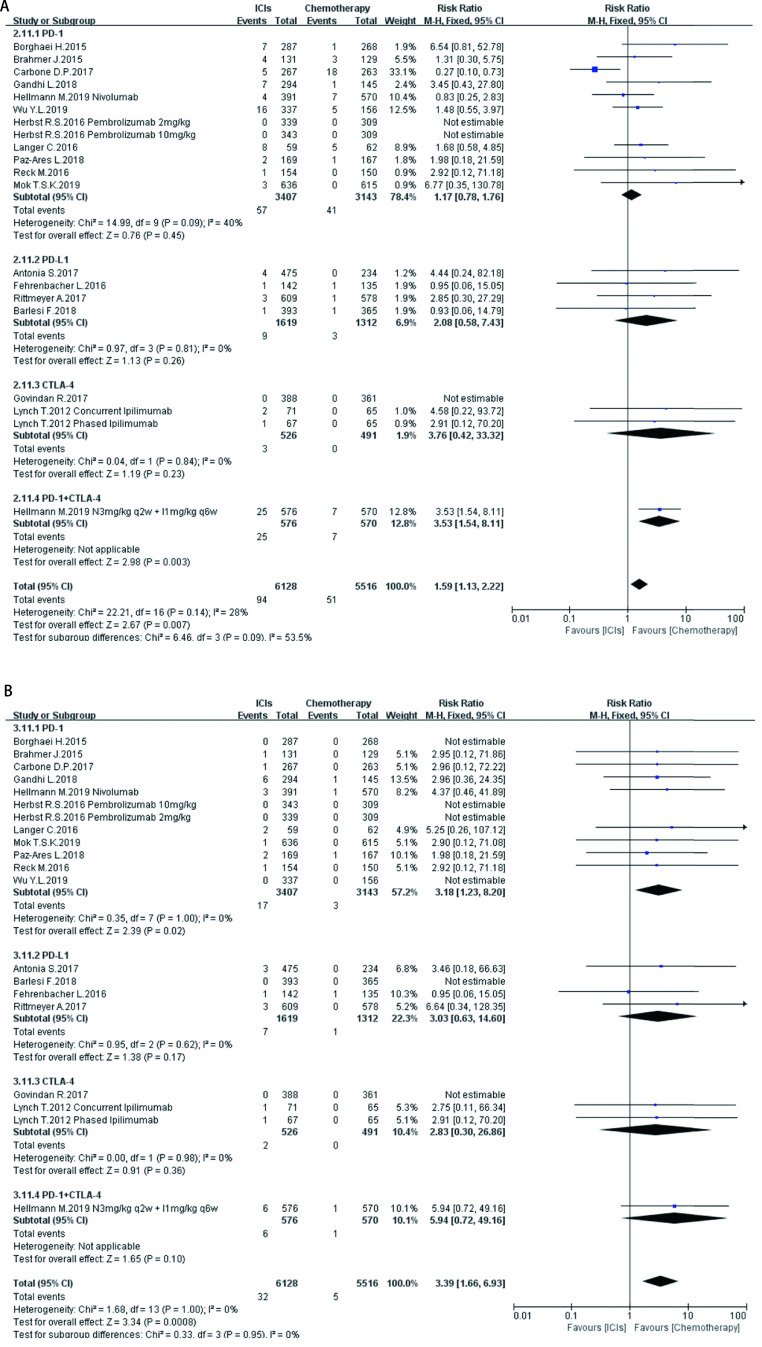
ICIs对比传统化疗所致肝脏ir-AEs。A：各级别肝脏ir-AEs; B：严重肝脏ir-AEs。 Hepatic ir-AEs induced by ICIs versus chemotherapy. A: Any grade hepatic ir-AEs; B: High grade hepatic ir-AEs.

**9:**
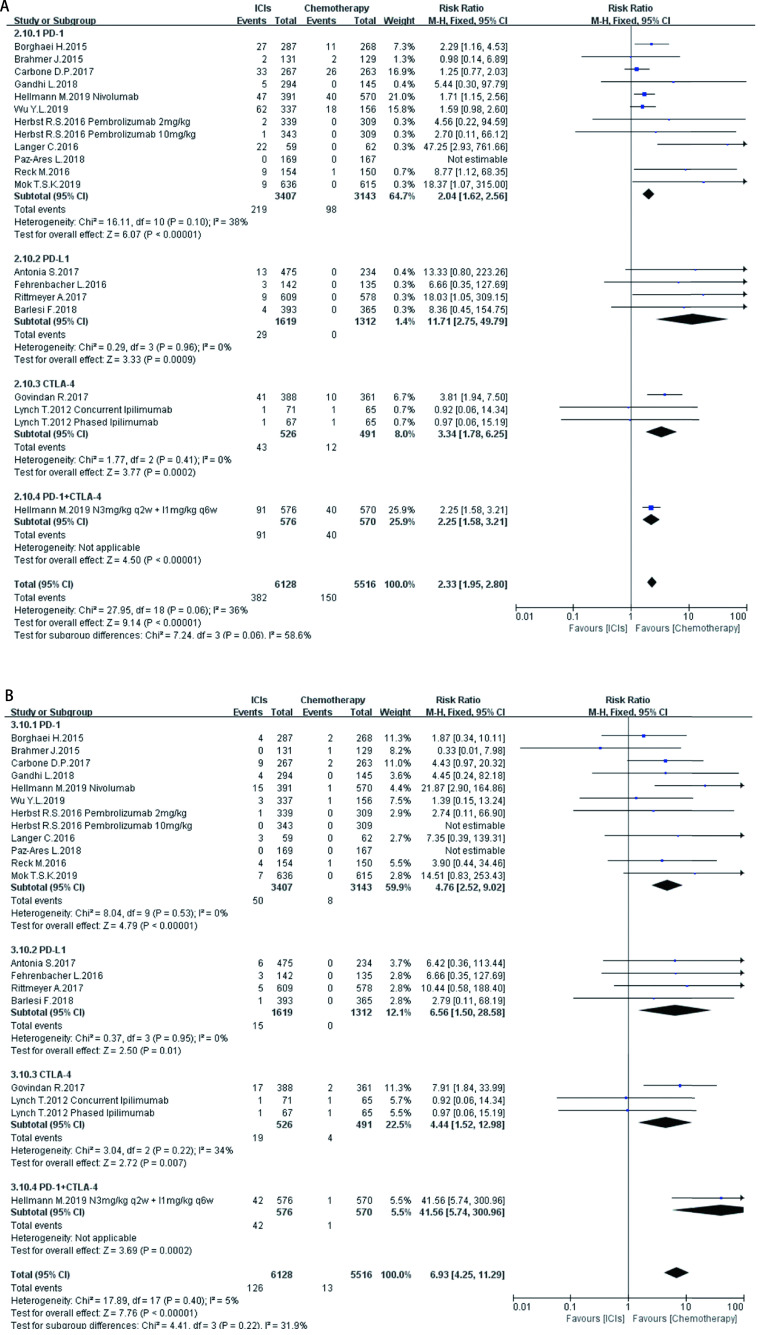
ICIs对比传统化疗所致肾脏ir-AEs。A：各级别肾脏ir-AEs; B：严重肾脏ir-AEs。 Renal ir-AEs induced by ICIs versus chemotherapy. A: Any grade renal ir-AEs; B: High grade renal ir-AEs.

**10:**
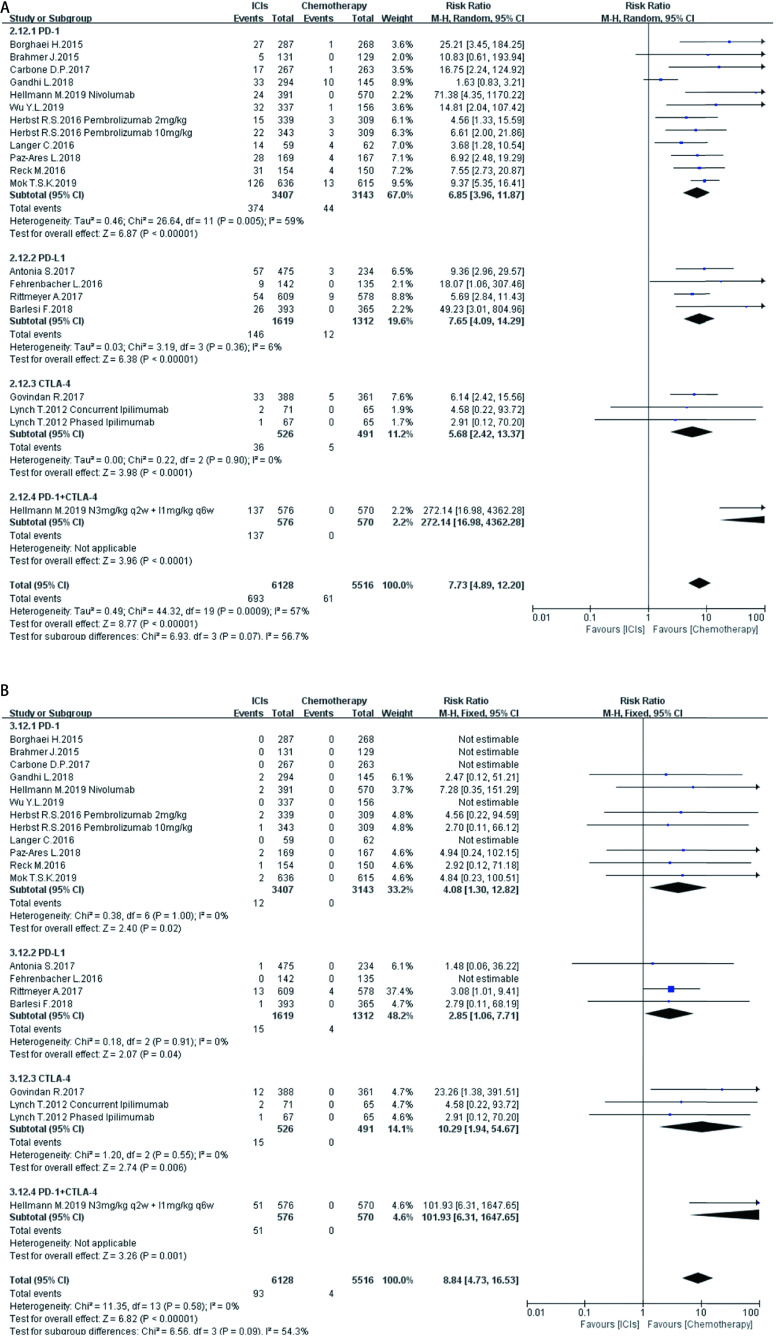
ICIs对比传统化疗所致内分泌ir-AEs。A：各级别内分泌ir-AEs; B：严重内分泌ir-AEs。 Endoctrine ir-AEs induced by ICIs versus chemotherapy. A:Any grade endoctrine ir-AEs; B: High grade endoctrine ir-AEs.

**11:**
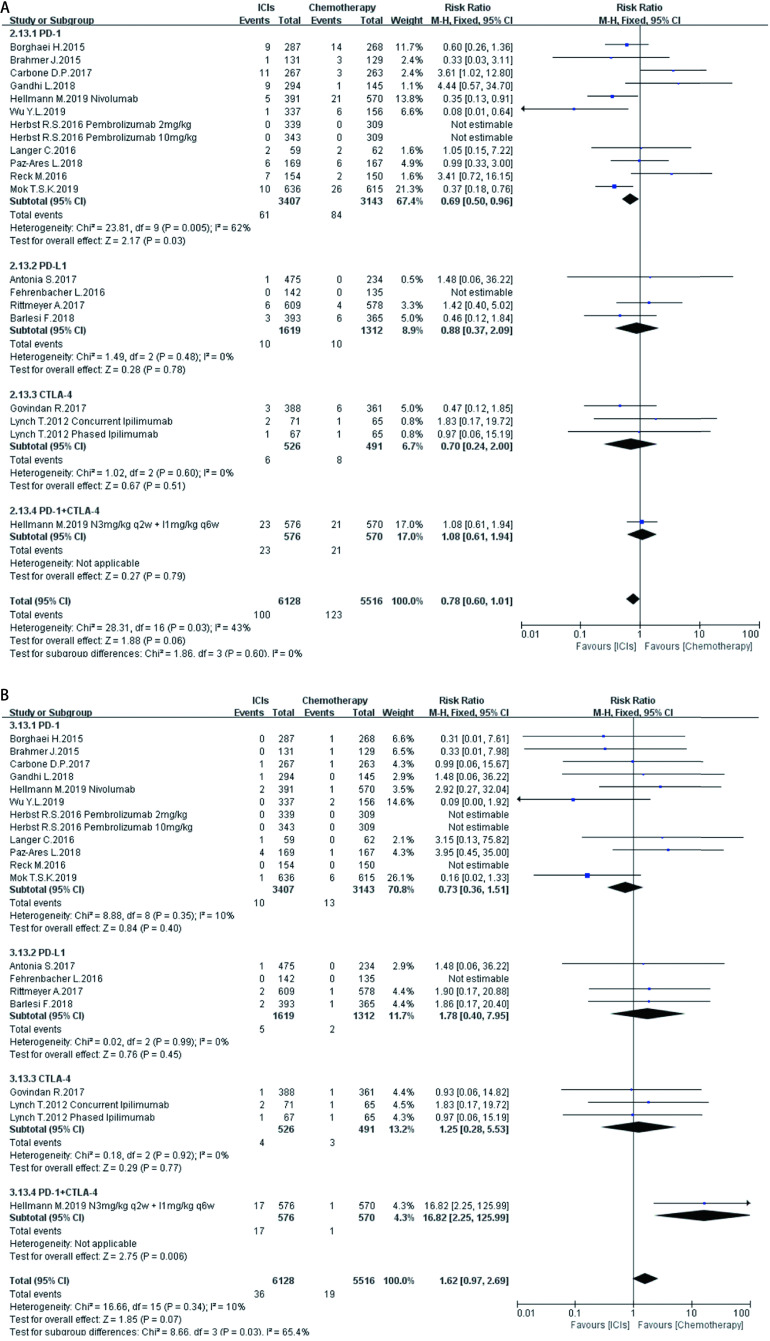
ICIs对比传统化疗所致过敏反应。A：各级别过敏反应; B：严重过敏反应。 Hypersensitivity induced by ICIs versus chemotherapy. A:Any grade hypersensitivity; B: High grade hypersensitivity.

在不同类型ICIs单药治疗的亚组分析中，我们发现不同ICIs对比传统化疗所致同一器官ir-AEs的发病风险存在差异：①皮肤：各ICIs单药治疗较传统化疗所致各级别免疫相关性皮炎的发病风险相近，其95%置信区间大部分重合差异无统计学意义; 抗CTLA-4对比传统化疗所致严重(3级及以上)免疫相关性皮炎的发病风险高于抗PD-1、PD-L1组(RR=5.31, 95%CI: 1.96-14.40, *P*=0.001)。②肺脏：抗PD-1组对比传统化疗所致各级别及严重免疫相关性肺炎的发病风险最高(RR=5.54, 95%CI: 3.06-10.02, *P* < 0.00001; RR=4.60, 95%CI: 2.67-7.92, *P* < 0.000, 1)。③胃肠道：抗PD-1、PD-L1对比传统化疗所致各级别及严重免疫相关性胃肠炎的发病风险无显著差异，抗CTLA-4所致各级别及严重免疫相关性胃肠炎的发病风险较传统化疗稍高(RR=1.83, 95%CI: 1.43-2.35, *P* < 0.000, 01; RR-3.90, 95%CI: 2.10-7.23, *P* < 0.000, 1)。④肝脏：抗PD-L1对比传统化疗所致各级别及严重免疫相关性肝炎的发病风险最高(RR-11.71, 95%CI: 2.75-49.79, *P*=0.0002; RR-6.56, 95%CI: 1.50-28.58, *P*=0.01)。⑤肾脏：抗PD-L1、CTLA-4对比传统化疗所致各级别及严重肾脏ir-AEs的发病风险无明显差异，抗PD-1所致严重肾脏ir-AEs的发病风险较传统化疗稍高(RR-3.18, 95%CI: 1.23-8.20, *P*=0.02)。⑥内分泌：3种ICIs对比传统化疗所致各级别内分泌ir-AEs的发病风险相近，差异无统计学意义; 抗CTLA-4组对比传统化疗所致严重内分泌ir-AEs的发病风险(RR=10.29, 95%CI: 1.94-54.67, *P*=0.006)明显高于抗PD-1、抗PD-L1组。⑦过敏反应：各ICIs对比传统化疗所致各级别及严重过敏反应的发病风险无明显差异。

同时，抗PD-1联合抗CTLA-4组所致ir-AEs的发病风险一般高于各单药治疗组。以各级别内分泌ir-AEs为例，抗PD-1联合抗CTLA-4组RR=272.14(95%CI: 16.98-4, 362.28, *P* < 0.000, 01)，而抗PD-1组、抗PD-L1组、抗CTLA-4组RR分别为6.85 (95%CI: 3.96-11.87, *P* < 0.000, 01)、7.65 (95%CI: 4.09-14.29, *P* < 0.000, 01)、5.68(95%CI: 2.42-13.37, *P* < 0.000, 1)。但值得注意的是，这一现象并不表现在所有器官上。例如，ICIs联合治疗对比传统化疗所致各级别胃肠道ir-AEs的RR为1.89(95%CI: 1.39-2.56, *P* < 0.000, 1)，抗CTLA-4组的RR为1.83(95%CI: 1.43-2.35, *P* < 0.000, 01)，两者95%CI大部分重合差异无统计学意义。

#### 2.2.2 不同ICIs所致ir-AEs的总体发病率

如表 2所示，我们对不同类型的ICIs所致ir-AEs进行亚组分析发现：①不同ICIs所致各级别ir-AEs的总体发病率：抗PD-1联合抗CTLA-4组所致各级别ir-AEs的总体发病率高于各单药治疗组，为12.2%(95%CI: 7.6%-19.6%); 单药治疗组中抗CTLA-4所致各级别ir-AEs的总体发病率最高(5.2%, 95%CI: 2.0%-13.4%)，其次为抗PD-1组(4.3%, 95%CI: 2.6%-7.0%)，抗PD-L1组的总体发病率最低(1.9%, 95%CI: 0.7%-4.9%)。②不同ICIs所致严重(3级及以上)ir-AEs的总体发病率：抗PD-1联合抗CTLA-4组所致严重ir-AEs的总体发病率最高，为4.9%(95%CI: 3.3%-7.2%); 单药治疗组中抗CTLA-4所致严重ir-AEs的总体发病率最高(2.1%, 95%CI: 1.0%-4.7%)，抗PD-1组(0.6%, 95%CI: 0.3%-1.1%)、抗PD-L1组(0.7%, 95%CI: 0.5%-0.9%)，两者95%CI大部分重合差异无统计学意义。

**2:** 各ICIs所致ir-AEs的总体发病率 The overall incidence of ir-AEs induced by ICIs

ICIs	All-grade ir-AEs incidence (%) (95%CI)	High-grade ir-AEs incidence (%) (95%CI)
PD-1	4.3 (2.6-7.0)	0.6 (0.3-1.1)
PD-L1	1.9 (0.7-4.9)	0.7 (0.5-0.9)
CTLA-4	5.2 (2.0-13.4)	2.1 (1.0-4.7)
PD-1+CTLA-4	12.2 (7.6-19.6)	4.9 (3.3-7.2)
Overall	5.1 (2.4-10.9)	1.4 (0.5-4.4)
ir-AEs: immune-related adverse events; ICIs: immune checkpoint inhibitors.		

#### 2.2.3 不同ICIs所致器官特异性ir-AEs的发病率

本文纳入的所有研究均报道了其观察到的器官特异性免疫相关不良事件。如图 12、图 13所示，我们对所报道的各器官特异性ir-AEs进行*meta*分析发现：①皮肤ir-AEs：在所有级别的器官特异性ir-AEs中，皮肤相关免疫不良事件发病率最高，为13.3%(95%CI: 9.8%-16.7%)，但鲜少发生严重ir-AEs，发病率仅1.2%(95%CI: 0.8%-1.6%)。其中，抗CTLA-4组(31.6%, 95%CI: 20.6%-42.6%)与联合治疗组(34.0%, 95%CI: 30.2%-37.9%)各级别免疫相关性皮炎的发病率最高，约为抗PD-1/PD-L1组的3倍。②肺脏ir-AEs：免疫相关性肺炎的发病率总体较低，为4.6%(95%CI: 3.2%-5.9%)，但通常较为严重，3级以上严重免疫相关性肺炎的发病率为1.6%(95%CI: 1.1%-2.2%)。单药治疗组中，抗PD-1组所致各级别免疫相关性肺炎的发病率最高，为5.6%(95%CI: 4.6%-6.5%)，严重者发病率低于2%;联合治疗组中免疫相关性肺炎的发病率较单药治疗组明显升高，其中各级别免疫相关性肺炎的发病率为8.3%(95%CI: 6.1%-10.6%)，3级以上免疫相关性肺炎的发病率为3.3%(95%CI: 1.8%-4.8%)。③胃肠道ir-AEs：在三种ICIs单药治疗组中，抗CTLA-4组免疫相关性胃肠炎的发病率最高(29.1%，95%CI: 25.2%-32.9%)，且高于抗PD-1联合抗CTLA-4组(18.2%, 95%CI: 15.1%-21.4%)。抗PD-L1所致各级别免疫相关性胃肠炎的发病率最低(0.5%, 95%CI: 0%-0.9%)，但通常较为严重(0.3%, 95%CI: 0%-0.6%)④肝脏ir-AEs：免疫相关性肝炎在单药治疗组中的发病率总体较低(低于10%)且鲜少发生严重ir-AEs; 但在抗PD-1联合抗CTLA-4组治疗组中发病率较高(15.8%, 95%CI: 12.8%-18.8%)，且3级以上不良事件的发病率高于该组所致的其他器官特异性严重ir-AEs(7.3%, 95%CI: 5.2%-9.4%)。⑤肾脏ir-AEs：免疫相关性肾炎的发病率在单药及联合治疗组中均低于5%，且严重者在各治疗组中的发病率均低于1%。⑥内分泌ir-AEs：在接受ICIs治疗的患者中，约10%的患者会出现各种类型的内分泌ir-AEs，其中以抗PD-1联合抗CTLA-4组(23.8%, 95%CI: 20.3%-27.3%)最为常见，但严重ir-AEs在各单药治疗组中的发病率均低于5%。⑦过敏反应：过敏反应在各ICIs治疗组中的发病率均较低(< 5%)，严重者发病率低于1%。

**12:**
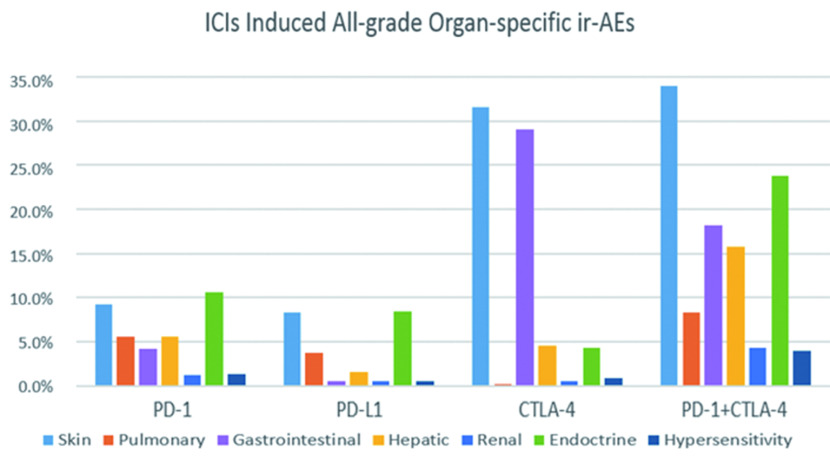
各级别器官特异性ir-AEs的发病率 The incidence of all-grade organ-specific ir-AEs

**13:**
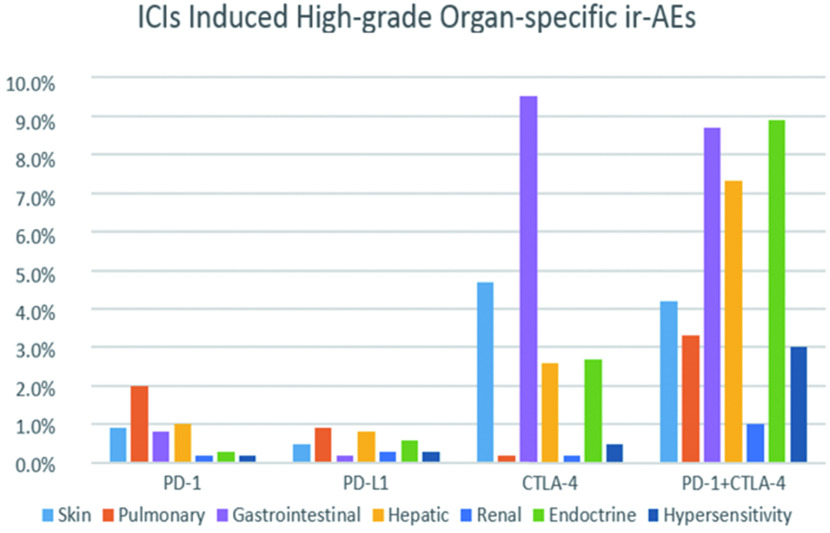
严重器官特异性ir-AEs的发病率 The incidence of high-grade organ-specific ir-AEs

## 3 讨论

近年来随着*EFGR*、*ALK*、*BRAF*、*KRAS*等驱动基因的发现，分子靶向治疗为具有驱动基因阳性突变的晚期NSCLC患者在延长生存上带来了切实获益。然而，我们应当认识到大多数NSCLC患者并不存在驱动基因的阳性突变。对于这些患者，其一线治疗方案的选择在过去仅限于细胞毒性化疗，而这种方案在延长生存上的疗效有限。与细胞毒性化疗、分子靶向治疗相比，免疫检查点抑制剂通过阻断PD-1/PD-L1、CTLA-4信号通路，解除肿瘤细胞的免疫耐受状态，从而产生抗肿瘤效应。自2011年FDA批准伊匹单抗(Ipilimumab, Yervoy^®^)上市以来，多种抗PD-1/PD-L1、CTLA-4免疫检查点抑制剂如纳武单抗(Nivolumab, Opdivo^®^)、派姆单抗(Pembrolizumab, Keytruda^®^)、阿特珠单抗(Atezolizumab, Tecentriq^®^)等也因其令人瞩目的疗效而备受关注。

在以PD-1、PD-L1、CTLA-4为靶点的免疫检查点抑制剂为晚期NSCLC患者的生存带来新希望的同时，其介导的新的免疫毒性ir-AEs，由于其特异性、严重性亦频发报道。在本研究中，我们系统的描述了ICIs治疗的晚期NSCLC患者所致irAEs的发生情况及影响因素，现就研究过程中发现的几点问题讨论如下。

### 3.1 ir-AEs的好发因素

在本研究中，我们观察到同样接受ICIs治疗的患者，一部分会出现严重的ir-AEs，而另一部分患者则未出现ir-AEs，这是为什么呢？一方面我们知道，在未接受ICIs治疗的患者中，某些自身免疫疾病的发生受基因影响较大^[23]^。有研究^[24]^汇总了453例接受Ipilimumab治疗黑色素瘤患者，来评价遗传因素与接受ICIs治疗的人群发生ir-AEs风险的相关性。该研究表明某种特定基因型与ir-AEs发生风险之间并无明确的相关性。故而，要明确遗传因素与ir-AEs发生风险之间的关系，下一步大规模的全基因组相关性分析是必需的。另一方面，Vetizou M等^[25]^的研究表明阻滞CTLA-4的ICIs的治疗有效性受肠道菌群组成(脆弱类杆菌和/或多形类杆菌和伯克氏菌)的影响。肠道菌群的组成通过影响白细胞介素12(interleukin-12, IL-12)依赖的Th1型免疫反应，抑制小鼠和患者体内肿瘤的生长。Sivan A等^[26]^的研究则发现单独口服双歧杆菌在抑制肿瘤生长上与PD-L1阻断治疗具有相同的疗效，而两者联合治疗则几乎完全抑制了肿瘤生长。研究还发现，树突状细胞功能的增强导致CD8^+^ T细胞在肿瘤微环境中的启动和聚集也得到增强，从而介导了这一作用。以上研究表明，肠道菌群的组成能够调控ICIs的疗效。由此我们推断，肠道菌群的变化会通过影响宿主的免疫力，从而影响接受ICIs治疗患者ir-AEs的发病风险。

### 3.2 ir-AEs的发生机制

那么ir-AEs又是怎么发生的呢？我们都知道免疫系统通过区分“自己”和“异己”，在维持稳态、保护宿主免受外源性病原体侵害方面发挥重要作用^[27]^。正常机体中，免疫系统通过免疫识别肿瘤细胞，并通过免疫反应将其清除，而肿瘤细胞则通过免疫编辑(包括清除、平衡、逃逸三个阶段)逃避免疫系统的攻击。在正常机体中，免疫耐受与免疫反应处于动态平衡中，但当这种平衡被打破，机体则会产生各种各样的疾病：如果出现免疫缺陷，机体将丧失自我防御能力无法识别“异己”，最终导致感染、肿瘤发生发展; 如果出现免疫耐受缺陷，机体固然可以消灭外源性病原体和肿瘤，但也会发生自身免疫性疾病，最终破坏自身组织和器官。

PD-1表达于B淋巴细胞、NK细胞以及激活的T细胞上，是一种免疫抑制分子，而多种肿瘤细胞可表达其配体PD-L1，PD-1与PD-L1结合从而抑制CD8细胞毒性免疫应答和由此产生的抗肿瘤免疫应答^[28]^，导致肿瘤免疫耐受。CTLA-4是一种白细胞分化抗原，是T细胞上的一种跨膜受体，与CD28共享B7分子配体，CTLA-4与抗原递呈细胞(antigen presenting cell, APC)的B7分子结合后可抑制T细胞活性，参与免疫反应的负性调节。因此，抗PD-1/PD-L1、CTLA-4单克隆抗体能够通过打破肿瘤细胞的免疫耐受，增强T细胞的抗肿瘤活性。目前的研究^[29]^表明，在肿瘤微环境中，PD-1、PD-L1、CTLA-4等免疫抑制分子存在明显的过表达现象。目前，FDA批准的ICIs主要通过阻断PD-1/PD-L1、CTLA-4信号通路，解除肿瘤细胞的免疫耐受状态，从而产生抗肿瘤效应。但由于其非特异性的正向免疫调节作用，打破了机体自身的免疫平衡状态，从而介导产生的免疫相关不良反应。这些不良反应涉及皮肤、消化系统、呼吸系统、泌尿系统、内分泌及代谢系统、骨骼及肌肉等诸多方面。

### 3.3 ir-AEs的发病特点

#### 3.3.1 ir-AEs的发生时间

在本研究中，我们发现接受ICIs治疗所致的ir-AEs的发生时间并不固定，它可以在接受ICIs治疗的任何时候出现，包括治疗过程中以及停止以后，但通常在治疗开始后数周至数月内发生，并随着病程的推移而发生波动。尽管接受ICIs治疗的持续时间可达数月甚至数年，但大多数研究^[30]^表明，长期暴露不会增加ir-AEs的累积发病率。然而，关于免疫检查点阻断是否会引起迟发型ir-AEs，由于纳入的相关前瞻性的临床试验尚在进行当中故目前尚不明确。

#### 3.3.2 ir-AEs的肿瘤特异性

此外，我们在进行文献筛选的过程中注意到一个有趣的现象：早在1964年Burdick等^[31]^的一项关于黑色素瘤的研究中就提到，接受免疫刺激治疗的患者白癜风的发病率明显升高。而本研究中对于接受ICIs治疗的晚期NSCLC患者，并未观察到类似现象。这提示ir-AEs的发生可能因肿瘤类型而异。而根据既往我们对抗肿瘤治疗毒性反应的认识，不同肿瘤类型的毒性反应通常极为相似。因此我们进一步推测，相较于ir-AEs的肿瘤特异性，不同ir-AEs的产生可能与抗原特异性免疫应答更为直接相关。

#### 3.3.3 不同ICIs的特异性ir-AEs

最近一项对恶性黑色素瘤患者的回顾性研究^[32]^显示，Ipilimumab所致严重ir-AEs经免疫抑制治疗缓解后，抗PD-1治疗可以安全地实施。这项研究提示，不同ICIs的免疫相关毒性谱可能是特异性的。本文中基于不同类型ICIs所致ir-AEs的亚组分析，进一步佐证了这个推测：接受抗CTLA-4治疗的患者与接受抗PD-1/PD-L1治疗的患者所发生的ir-AEs不尽相同，而且抗CTLA-4治疗产生的ir-AEs通常更多且更为严重。例如，免疫相关结肠炎在抗CTLA-4治疗中更为常见，而自身免疫性肺炎和自身免疫性甲状腺炎则更常见于抗PD-1治疗。尽管这两种ICIs免疫相关毒性反应存在差异的原因尚不明确，我们推测这可能与不同类型ICIs激活免疫系统的通路不同有关。此外在关于自身免疫性甲状腺炎的报告中，人们发现，在甲状腺自身抗体阳性(无论治疗前基线水平是否为阳性)并接受抗PD-1治疗的患者中，发生自身免疫性甲状腺炎的风险较高。可能的解释是除了T细胞介导的免疫反应外，抗PD-1或抗PD-L1治疗还可调节体液免疫反应，从而使基线存在的甲状腺自身抗体的水平升高^[4]^。

此外，虽然目前各ICIs单药或联合治疗所致各个器官ir-AEs的发病率尚缺乏完善的统计学资料，但本研究表明抗PD-1与抗CTLA-4联合治疗所致ir-AEs的发病率一般高于单药治疗组，尤其在内分泌ir-AEs及免疫相关性肺炎上表现最为显著，但免疫相关性结肠炎及肝炎除外。这提示ICIs单药治疗晚期NSCLC患者较联合治疗耐受性更好，但两者所产生抗肿瘤效应的差异对患者OS、PFS、ORR的影响尚不明确。因此ICIs联合治疗对晚期NSCLC患者带来的获益及风险尚需进一步的临床试验加以明确。

#### 3.3.4 ir-AEs的器官特异性

在统计过程中我们发现，ICIs所致ir-AEs可累及全身各个器官，包括皮肤、胃肠道、肝脏、肾脏、肺脏、内分泌器官，甚至中枢神经系统等。下文就ICIs治疗晚期NSCLC所致各器官特异性ir-AEs做一系统介绍。

##### 3.3.4.1 皮肤

本研究发现，皮肤ir-AEs是ICIs单药或联合治疗过程中最常见的ir-AEs，但3级以上者鲜少发生。其中，又以皮疹、瘙痒、红斑、白癜风最为多见。对轻度(< 3级)皮肤ir-AEs，糖皮质激素与抗敏止痒药物联合外用疗效确切，但在应用激素治疗前需先除外感染因素。对于严重(≥3级)ir-AEs，则需先行皮肤活检明确病变组织学类型，并全身系统应用糖皮质激素治疗。

##### 3.3.4.2 肺脏

免疫相关性肺炎包括结节病、间质性肺炎、局限性肺炎等，本研究发现虽然其发病率较低，但通常较为严重甚至危及生命，因此要求临床医生警惕患者在免疫治疗过程中出现的进行性气短、干咳等临床表现。若怀疑免疫相关性肺炎，需行胸部CT及肺功能检查。典型的免疫相关性肺炎影像学表现为磨玻璃影、局灶和/或播散性结节浸润，尤以下叶为著^[3]^。为明确感染病原体，需行支气管镜及支气管肺泡灌洗。其治疗需全身应用激素治疗，但倘若应用激素治疗后初始症状未缓解，则需考虑使用抗肿瘤坏死因子α(anti-tumor necrosis factor α, anti-TNF-α)抗体英夫利昔单抗(Infliximab)进行免疫抑制治疗。

##### 3.3.4.3 胃肠道

免疫相关性胃肠炎一般表现为腹痛、腹泻等非特异性消化系统症状，本研究发现在接受各种不同类型ICIs治疗的患者中，尤以抗CTLA-4组所致免疫相关性胃肠炎的发病率(29.1%, 95%CI: 25.2%-32.9%)为高。其诊断需依赖内镜检查及病理活检，镜下可见黏膜红斑、溃疡、隐窝脓肿和肉芽肿等，与克罗恩病的镜下表现相似; 且需行便培养与感染性腹泻相鉴别、行粪便钙卫蛋白检测与功能性肠病相区分; 此外，还需行腹部计算机断层扫描显像(computed tomography, CT)评估胃肠病变的严重程度和累及范围。在治疗上，轻度(1级-2级)免疫相关性胃肠炎可用布地奈德治疗，而严重(≥3级)者则需全身应用糖皮质激素治疗，持续不缓解者(全身足量激素治疗3天症状无减轻)可考虑短期应用英夫利昔(infliximab)治疗^[33]^。

##### 3.3.4.4 肝脏

本研究中，免疫相关性肝炎的总体发病率不高(5.1%, 95%CI: 3.6%-6.5%)，但通常较为严重(1.5%, 95%CI: 0.9%-2.0%)。免疫相关性肝炎常表现为无症状性肝酶升高，故临床医生需警惕接受免疫治疗患者的实验室检测结果异常(AST、ALT升高)，同时需排除病毒感染相关肝炎。免疫相关性肝炎的血清学抗体(抗核抗体、抗平滑肌抗体、抗肝肾微粒体抗体-1等)检测常为阴性，其确诊有赖于肝穿刺活检^[34]^。确诊患者的管理可参照自身免疫性肝炎，对于激素难治性病例，可加用氮唑嘌呤或吗替麦考酚酯。

##### 3.3.4.5 肾脏

肾脏ir-AEs的发病率总体较低，其病理类型主要包括间质性肾炎、肉芽肿性肾炎、肾小球狼疮样肾病^[35]^，可以无症状性血尿、蛋白尿为首发表现，故需定期行尿常规、尿微量蛋白等相关化验检查。同样地，其确诊也有赖于肾穿刺活检，确诊患者的管理以全身应用糖皮质激素为主。

##### 3.3.4.6 内分泌系统

本研究的统计分析发现内分泌ir-AEs的总体发病率较高(9.9%, 95%CI: 7.4%-12.3%)，仅次于皮肤ir-AEs，主要包括：甲状腺功能减退、甲状腺功能亢进、垂体炎、肾上腺功能不全等。其中甲状腺功能减退比甲状腺功能亢进更常见。而甲状腺功能亢进几乎在所有病例中都是自发消退的^[36]^，随后出现甲状腺功能减退对于甲减患者，应监测促甲状腺激素(thyroid stimulating hormone, TSH)、血清游离三碘甲腺原氨酸(fT3)、血清游离甲状腺素(fT4)水平，并及时开始激素替代治疗(如左甲状腺素)。对于内分泌ir-AEs，通常不建议使用糖皮质激素治疗，因为这可能会加重代谢功能障碍。

##### 3.3.4.7 神经系统

本文中因纳入的多项RCTs对神经系统ir-AEs未作报道，故在此仅对其做一般性描述。ICIs所致神经系统ir-AEs主要包括：吉兰巴利综合征、无菌性或淋巴细胞性脑膜炎、可逆性后部脑病综合征、横贯性脊髓炎等^[37]^。其报道主要见于接受Ipilimumab治疗的患者，而接受抗PD-1/PD-L1治疗的患者中则鲜少报道。神经系统ir-AEs相对较严重，一旦发生常需立即停药并全身应用糖皮质激素治疗。

### 3.4 ir-AEs的管理

目前临床上对ir-AEs的管理一般基于基于药物的安全性报告^[38]^、专家共识^[39]^以及既往对自身免疫性疾病的认识。虽然目前ir-AEs发病的确切病理生理机制尚不明确，但可以肯定的是是由免疫系统对正常器官的过度免疫反应引起的。因此，我们可以通过推迟给予ICIs，或者对于较为严重的病例，给予口服糖皮质激素或其他免疫抑制剂从而诱导一过性的免疫抑制，大多数ir-AEs均可得到有效控制。许多报告介绍了基于临床经验的处理流程，并为如何治疗特定的ir-AEs提供了详细的实践指导。

大多数ir-AEs是激素敏感的，并可在6周-12周内缓解^[40]^。应用激素时应起始足量，但应用时间不应过长，以避免降低ICIs的抗肿瘤作用。足量激素治疗2周-4周后，可开始缓慢减量(至少1个月)，以避免ir-AEs复发。同时为预防机会性感染等激素相关不良事件，常需应用复方磺胺甲恶唑等激素保驾药物。

当发生激素难治性ir-AEs时，可以尝试免疫调节剂或免疫抑制剂，如TNF-α拮抗剂、硫唑嘌呤和吗替麦考酚酯等^[39]^。其中抗TNF-α起效较快，而其他免疫抑制剂，如硫唑嘌呤和MMF通常在几周后才会起效。英夫利西单抗是一种TNF-α拮抗剂，用于克罗恩病和溃疡性结肠炎的治疗; 有研究^[41]^表明，其对ICIs诱发的中重度结肠炎患者也有疗效。在ir-AEs的治疗过程中，如果糖皮质激素治疗不成功，通常建议使用英夫利西单抗。但是，考虑到英夫利西单抗有可能快速起效，以及糖皮质激素长期治疗的毒性反应，那么，在治疗免疫相关不良事件时，为了尽量降低糖皮质激素的暴露，英夫利西单抗是否应该更早使用？这一问题有待更多临床经验以进一步明确。

### 3.5 ICIs治疗晚期NSCLC患者的临床结局与ir-AEs的关系

虽然目前ir-AEs发生的确切病理生理机制尚不明确，但可以肯定的是与ICIs激活免疫系统，打破原有的免疫平衡有关。而这种免疫激活效应是否与抗肿瘤效应的增强有关，至今仍然存在争议。Downey等^[42]^的研究显示，出现ir-AEs患者的客观缓解率高于未出现ir-AEs的患者。Horvat等^[43]^的研究则显示出现ir-AEs的患者与未出现患者的临床结局相似。故免疫系统的激活是否会增加患者的客观缓解率，ir-AEs的严重程度是否可以成为抗肿瘤免疫效应的一个衡量指标，还有待于进一步的临床试验进行证实。至少目前的基本共识认为，接受ICIs治疗患者的临床获益无须此类ir-AEs。

我们的研究有一些局限性。首先，临床试验方案的可变性很高。这是本研究异质性的一个重要来源，因此我们对ir-AEs等级、器官特异性、ICIs类型进行亚组分析，这样异质性的主要来源显著减少。这些亚组分析的结果从另一方面来讲为临床医生在使用不同的ICIs方案治疗晚期NSCLC患者时警惕ir-AEs的发生提供了临床参考。其次，根据药物类型、器官特异性进行亚组分析的患者数量有限。并且，由于接受抗PD-L1和抗CTLA-4的几项RCTs仍在进行中，ir-AEs报告不完整，故未纳入本研究，这导致对于ICIs联合治疗晚期NSCLC患者的疗效及ir-AEs分析缺乏这部分的数据支持。第三，纳入研究对象的治疗基线不同，这在我们的分析中引入了一些偏倚。最后，根据我们的分析，在存在自身免疫性疾病的患者中，ir-AEs的发病率并不清楚。因为所有纳入的研究都排除了已患有自身免疫性疾病或既往有行糖皮质激素或免疫抑制剂治疗病史的患者。因此，ICIs在已有自身免疫性疾病患者中的安全性需要在实验或临床研究中进一步评估。

综上所述，ICIs相较于传统化疗具有不同的毒性谱，其所致ir-AEs一般多于传统化疗，但鲜少发生严重ir-AEs。不同ICIs所致ir-AEs的发病率不同，且具有器官特异性。抗CTLA-4较抗PD-1/PD-L1治疗所致ir-AEs的总体发病率更高。ICIs联合治疗所致ir-AEs的总体发病率一般高于单药治疗。

随着ICIs逐渐成为治疗晚期NSCLC的一种新型、有效的治疗方案，我们的*meta*分析提供了接受抗PD-1/PD-L1/CTLA-4治疗的NSCLC患者的ir-AEs的发生概况。在今后的临床试验中应进一步评价ICIs所致迟发型ir-AEs的发病特点、ir-AEs对接受ICIs治疗患者生活质量的影响、免疫抑制剂治疗ir-AEs对ICIs抗肿瘤作用的影响，以及ICIs对潜在自身免疫性疾病的晚期NSCLC人群的安全性。同时，ICIs治疗晚期NSCLC患者所致ir-AEs的管理通常需要多学科之间的相互协作。随着晚期NSCLC迎来免疫治疗的新时代，通过对ICIs治疗晚期NSCLC患者疗效及ir-AEs的深入探索及认识，我们相信更多晚期NSCLC患者会从免疫疗法中获益。

**Author contributions**

Qin QX and Wang H conceived and designed the study. Qin QX and Wang JJ performed the experiments. Qin QX and Wang JJ analyzed the data. Qin QX and Wang JJ contributed analysis tools. Qin QX, Wang JJ and Wang H provided critical inputs on design, analysis, and interpretation of the study. All the authors had access to the data. All authors read and approved the final manuscript as submitted.
